# Long-Term Adherence and Persistence to Low-Dose Aspirin for the Prevention of Cardiovascular Disease: A Population-Based Cohort Study

**DOI:** 10.1155/2022/7786174

**Published:** 2022-12-02

**Authors:** Pareen Vora, Montse Soriano-Gabarró, Beth Russell, Henry Morgan Stewart

**Affiliations:** ^1^Integrated Evidence Generation, Bayer AG, Berlin, Germany; ^2^Comprehensive Cancer Centre, Kings College London, London, UK; ^3^IQVIA, Brighton, UK

## Abstract

**Methods:**

Using information from electronic health records in Germany and the United Kingdom (UK) in a common data model, we followed adults with ≥2 low-dose aspirin prescriptions (75–100 mg) during 2007–2018 for up to 10 years. Included individuals had no low-dose aspirin prescriptions in the year before the follow-up started (date of first low-dose aspirin prescription) and ≥12 months' observation. Adherence was determined using the medication possession ratio (MPR), and persistence was defined as continuous treatment disregarding gaps between prescriptions of <60 days; analyses were undertaken according to indication (primary/secondary CVD prevention).

**Results:**

We identified 144,717 low-dose aspirin users from Germany and 190,907 from the UK. Among patients with 5–10 years' follow-up, median adherence among secondary CVD prevention users was 60% in Germany and 75% in the UK. Among primary prevention users, median adherence was 50% for both countries. Persistence among secondary CVD prevention users was 58.3% at 2 years, 47.0% at 5 years, 35.2% at 10 years (Germany), and 67.5% at 2 years, 58.0% at 5 years, and 46.8% at 10 years (UK). Among primary CVD prevention users, persistence was 52.8% at 2 years, 41.6% at 5 years, 32.1% at 10 years (Germany), 56.3% at 2 years, 45.4% at 5 years, and 33.8% at 10 years (UK).

**Conclusions:**

Long-term adherence and persistence to low-dose aspirin are suboptimal; efforts for improvement could translate into a lower CVD burden in the general population.

## 1. Introduction

Long-term treatment with low-dose aspirin remains the foundation of pharmacotherapy for the secondary prevention of cardiovascular disease (CVD), with 12 months of concomitant use of a P2Y12 receptor inhibitor (dual antiplatelet therapy [DAPT]) indicated following acute coronary syndrome (ACS) [1–3]. The Antithrombotics Trialists' Collaboration meta-analysis of data from secondary prevention randomized controlled trials (RCTs) showed an approximate 20% reduced risk of major coronary events and stroke and a 13% reduced risk of vascular death in participants allocated to low-dose aspirin vs. control [4]. A recent meta-analyses of RCT data showed that low-dose aspirin is associated with an 11% reduction in cardiovascular outcomes (based on a composite cardiovascular endpoint) vs. control, yet no reduction in all-cause mortality was seen [5]. Low-dose aspirin is not indicated for primary CVD prevention in most countries, but it is still used in clinical practice for patients at high risk of CVD, albeit evidence suggests a decline in use over the last decade [6].

Adherence and persistence to low-dose aspirin are key to maximising its cardiovascular benefits in patients with a history of CVD or those at high CVD risk, and due to the chronicity of CVD and its precursors, life-long therapy is needed. This can be challenging for the patient, especially those taking low-dose aspirin for primary prevention purposes who have not yet experienced a major cardiovascular event. Several studies have shown that patients with poor adherence and those who discontinue their low-dose aspirin treatment are at increased risk of adverse cardiovascular outcomes compared with those who adhere/are persistent with treatment [7–12]. Population-based estimates of adherence and persistence to low-dose aspirin help inform clinical guiding dialogue with patients to encourage them to continue with their medication as directed. Estimates have been reported by many studies [9, 13–19] yet few of these have described long-term (up to 2–3 years) adherence and persistence. Furthermore, estimates specific to the type of user (primary or secondary CVD prevention) from the same study population are lacking, and many studies have been limited in size. Quantifying long-term aspirin adherence and persistence requires data sources that enable the long-term follow-up of large population-based cohorts and which are valid for pharmacoepidemiologic research. Using two such data sources from Germany and the United Kingdom (UK) and applying a standardised methodology, we aimed to describe long-term adherence and persistence (up to 10 years) to low-dose aspirin in these two Western European countries. The primary objective was to determine long-term adherence and persistence to low-dose aspirin (75–100 mg) in primary and secondary CVD prevention populations. The secondary objective was a subanalysis among patients who initiated low-dose aspirin as part of DAPT, including an evaluation of switching to low-dose aspirin monotherapy.

## 2. Methods

### 2.1. Study Design and Data Sources

This was a population-based cohort study set in routine clinical practice using longitudinal, anonymised electronic health records (EHRs) from the IQVIA Germany Disease Analyzer database [20, 21] and the IQVIA Medical Research Database-UK (IMRD-UK) database (formerly known as the Health Improvement Network). Both have previously been converted to a standardised common data model (CDM) format developed by the Observational Medical Outcomes Partnership (OMOP) (a public-private partnership established to inform the appropriate use of observational healthcare databases for studying the effects of medical products) which is updated by the Observational Health Data Sciences and Informatics (OHDSI) multistakeholder, interdisciplinary collaboration. With CDM, the specific coding system of each database is converted into systematized nomenclature of medicine (SNOMED), which allows the analysis of data from different databases in a standardised manner [22–24]. The IQVIA Germany Disease Analyzer database contains demographic, primary care, and outpatient medical data and prescriptions issued for a representative sample of approximately 38.5 million from about 2498 general practices across Germany. The IMRD-UK database contains records for approximately 17 million patients in the UK (∼6% of the UK population) and similarly includes demographic, medical, and prescription data entered as part of routine primary care, with information received from hospital visits entered retrospectively [25]. Both databases are representative of the wider population demographic and have been validated for use in pharmacoepidemiological research. [21, 26, 27] As this was a noninterventional observational study using secondary data, ethical approval was not required for the use of the Germany Disease Analyzer—IQVIA has an existing agreement for this database to be used for publication purposes. For analysis of the data from IMRD-UK, the study protocol was approved by an independent scientific research committee (SRC Reference Number: 19THIN88). Data collection for IMRD UK was approved by the South East Multicentre Research Ethics Committee in 2003 and individual studies using IMRD-UK data do not require separate ethical approval if only anonymised data are used.

### 2.2. Source Populations and Study Cohorts

Individuals aged ≥18 years with at least two prescriptions for low-dose aspirin (75–100 mg) between January 1, 2007, and December 31, 2018, were included; the date of the first prescription was the index date. Patients were required to have no prescription for low-dose aspirin in the 12 months before the index date and to have at least 12 months of observation before and after the index date. Individuals with missing data on age or sex were excluded.

The secondary CVD prevention cohort comprised all patients with a record of CVD (myocardial infarction, ischaemic stroke, transient ischaemic attack, unstable angina, angina, and ischaemic heart disease) or coronary artery bypass graft/percutaneous coronary intervention before the index date and/or a prescription for an alternative antiplatelet (e.g., clopidogrel, ticagrelor, prasugrel) before the index date. All remaining patients comprised the primary CVD prevention cohort. From within the secondary CVD prevention cohort, we identified a subcohort of patients starting on DAPT, defined as those with a prescription for clopidogrel, prasugrel, or ticagrelor within 30 days of the index date. Additionally, from within the primary prevention cohort, we defined a subcohort of patients with at least one of the following CVD risk factors at any time before the index date: diabetes mellitus, hyperlipidaemia, hypertension, obesity (body mass index (BMI) ≥30 kg/m^2^), smoking, or aged >50 years. No imputation was made for patients with missing data on BMI or smoking. Patients were followed from the date of their first low-dose aspirin prescription (index date) until the date of death, transfer out of the practice, or the end of the study period (maximum of 10 years from the index date), whichever came first.

### 2.3. Low-Dose Aspirin Adherence and Persistence

Low-dose aspirin prescription data was used to determine adherence and persistence. The number of days' supply was calculated using the prescribed pack size and physician-instructed daily dose. We considered a low-dose aspirin prescription to be “active” (assumed as taken) from the day the prescription was issued/dispensed until the end of supply. We calculated adherence for each patient using the medication possession ratio (MPR), defined as the number of supplied days of medication within the observation period divided by the number of days in the observation period.

**Figure 1:**
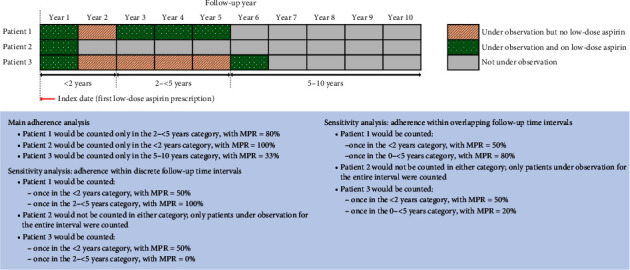
Adherence methodology: main analysis and sensitivity analyses by (a) discrete follow-up time intervals, (b) overlapping follow-up time intervals.

Overall adherence (over the entire follow-up period) was stratified according to patients' total length of follow-up: <2 years, >2 to 5 years, or >5 to 10 years (capped at 10 years) (Figure 1). Persistence was defined as no gaps of 60 days or more between the end of the supply of a low-dose aspirin prescription and the start of the following consecutive low-dose aspirin prescription. Longer consecutive treatment gaps were counted as multiples of 60. For example, 120–179 day treatment gaps were considered as two gaps, 180–239 gaps were considered as 3 treatment gaps, and so on. For the secondary CVD prevention DAPT cohort, we evaluated switching from DAPT to antiplatelet monotherapy.

### 2.4. Statistical Analysis

Summary statistics were used to describe patients' demographics at baseline. Cohort-level adherence and persistence were expressed as percentages. Adherence was summarised by calculating the median with the interquartile range (IQR). Persistence was calculated as the number of patients under observation with an active low-dose aspirin prescription in the last 60 days divided by the number of patients under observation. Further, persistence was evaluated according to the number of consecutive treatment gaps during therapy. We performed several sensitivity analyses. Firstly, we calculated adherence during discrete follow-up periods (<2 years, 2 to 5 years, and >5 to 10 years) rather than during the entire follow-up period; for this, only patients who were observable for the entirety of the discrete time period were included for the analysis of that time period, and patients could potentially contribute to more than one discrete time period (see example in Figure 1). Secondly, we calculated adherence within the following overlapping intervals of follow-up time: 0–2 years, 0–5 years, and 0–10 years, again only including patients who were observable for the entire time interval as appropriate (patients could also potentially contribute to more than one time period in this analysis; see example in Figure 1). Thirdly, we redefined adherence as the proportion of days covered (PDC); this was defined as the number of days in the time interval covered by an active low-dose aspirin prescription divided by the number of days in the time period. Fourthly, for persistence, we changed the definition of a gap in therapy from ≥60 days between consecutive active low-dose aspirin prescriptions to ≥30 days.

## 3. Results

We identified a total of 144,717 low-dose aspirin users from the IQVIA Germany Disease Analyzer database and 190,907 low-dose aspirin users from the IMRD-UK database. In Germany, 43,013 (30%) were in the primary CVD prevention cohort, and 101,704 (70.3%) were in the secondary CVD prevention cohort. In the UK, 72,496 (38.0%) were in the primary prevention cohort, and 118,411 (62%) were in the secondary prevention cohort. Ischaemic heart disease was the most prevalent type of CVD in the secondary prevention cohorts (59% in Germany, 38% in the UK; Supplementary Table 1). Patients initiating low-dose aspirin as part of DAPT accounted for 23% of the secondary prevention cohort in both countries. Among the primary prevention cohorts, 97% (Germany; *n* = 41,875) and 90% (UK; *n* = 65,090) had CVD risk factors recorded.

The demographics of the study cohorts are shown in Table 1; each had a median observation time of at least 4 years. The German cohorts were slightly older than the respective UK cohorts: 69.5 years vs. 65.0 years for primary CVD prevention, 69.0 years vs. 65.5 years for secondary CVD prevention, and 65.3 years vs. 63.1 years for the secondary prevention DAPT subcohort. The distribution of the sexes was roughly equal in the primary prevention cohorts, while males accounted for the majority of the secondary prevention cohorts, especially the DAPT subcohort (68% in Germany and 69% in UK). The primary prevention with risk factors subcohorts were, on average, the oldest (mean age, 70.3 years in Germany and 68.4 years in the UK) (Supplementary Table 2).

### 3.1. Adherence

**Figure 2:**
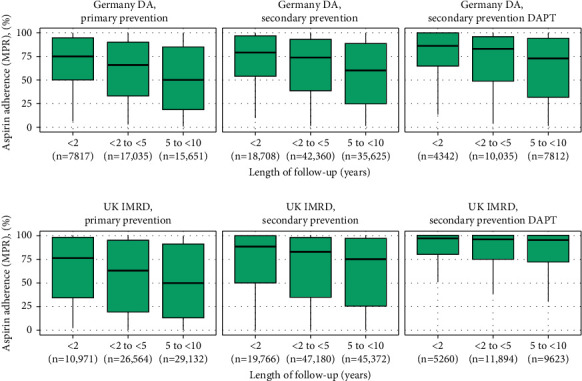
Low-dose aspirin adherence (MPR) over entire follow-up stratified by length of follow-up. DAPT, dual antiplatelet therapy; DA, disease analyzer; IMRD, IQVIA Medical Research Data; MPR, medication possession ratio; UK, United Kingdom.

Among patients with 5–10 years of available follow-up, median adherence in the secondary CVD prevention cohort was 60% in Germany (73% in the DAPT group) and 75% in the UK (95% in the DAPT group); in the primary CVD prevention cohort, median adherence among patients with 5–10 years of available follow-up was 50% in both countries (Figure 2; Supplementary Table 3a), with similar estimates among those with CV risk factors (Supplementary Table 3b).

### 3.2. Sensitivity Analyses

In the analysis of adherence during discrete follow-up periods, median adherence was highest in the first 2 years of follow-up and decreased over time (Supplementary Figure 1; Supplementary Tables 4a and 4b). For example, in the secondary prevention German cohort, median adherence was 82% in the first two years, dropping to 61% during the 2 to <5 years follow-up period, and 33% during the 5 to <10 years follow-up period; the corresponding proportions for the UK were 89%, 81%, and 58% (Supplementary Table 4a). In the primary prevention cohorts, fewer than 1 in 5 patients remained adherent to low-dose aspirin at 5 to <10 years' follow-up (in both countries), with similar proportions seen among those with CV risk factors (Supplementary Table 4b). In the analysis of adherence calculated during overlapping follow-up periods (0 to <2 years, 0 to <5 years and 0 to <10 year), estimates were, as expected, higher in the early years of follow-up. For example, in Germany, the median adherence in the secondary CVD prevention cohort was 82% during 0 to <2 years' follow-up, 68% during 0 to <5 years' follow-up, and 49% during 5 to <10 years' follow-up (Supplementary Figure 2; Supplementary Table 5). In the sensitivity analysis, where adherence was measured using PDC, estimates were slightly lower than those using MPR (Supplementary Figure 3).

### 3.3. Persistence

**Figure 3:**
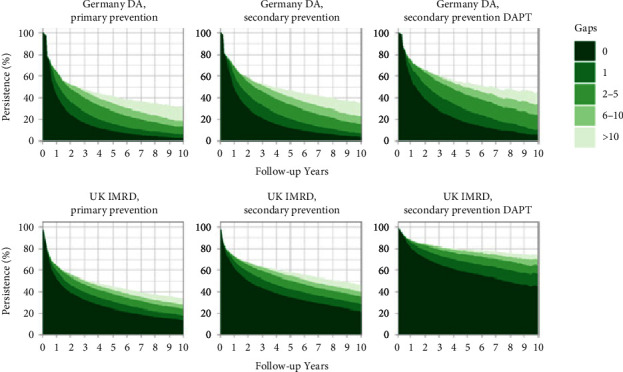
Low-dose aspirin persistence over time, stratified by number of consecutive 60 day gaps in therapy. DAPT, dual antiplatelet therapy; DA, disease analyzer; IMRD, IQVIA Medical Research Data; UK, United Kingdom.

Persistence in the German secondary CVD prevention cohort, irrespective of gaps in treatment, was 58% at 2 years, 47% at 5 years, and 35% at 10 years; corresponding proportions for the UK were higher at 68%, 58%, and 47% (Figure 3 and Supplementary Table 6). Persistence at 10 years in the DAPT cohort was 45% in Germany and 75% in the UK. In the primary CVD prevention German cohort, irrespective of gaps in therapy, persistence was 53% at 2 years, 42% at 5 years, and 32% at 10 years; corresponding data for the UK was similar at 56%, 45%, and 34%, respectively. Among patients with no gaps in therapy (i.e., regular returners for repeat prescriptions), persistence at 2 years in the German and UK cohorts, respectively, was 24% and 40% (primary CVD prevention), 30% and 51% (secondary CVD prevention), and 39% and 71% (secondary CVD DAPT prevention). Corresponding proportions in the German and UK cohort at 5 years (for patients with no gaps in therapy) were 8% and 24% (primary CVD prevention), 12% and 35% (secondary CVD prevention), and 17% and 58% (secondary CVD DAPT prevention), and at 10 years, they were 2% and 14% (primary CVD prevention), 3% and 23% (secondary CVD prevention), and 5% and 46% (secondary CVD DAPT prevention). In all study cohorts, there was a clear trend toward stable persistence following an initial drop and a trend toward an increasing number of consecutive gaps with time (i.e., increasing length of the break in treatment). Analysis based on gaps in treatment of ≥30 days, produced very similar findings to the main persistence analysis (Supplementary Figure 4; Supplementary Table 7).

### 3.4. Switching from DAPT to Antiplatelet Monotherapy

**Figure 4:**
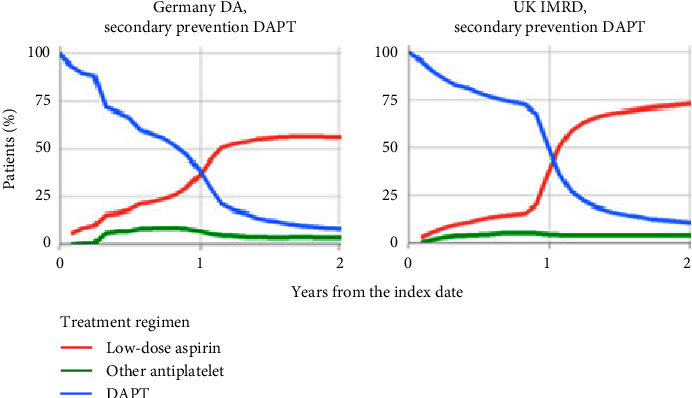
Antiplatelet use among patients in the DAPT secondary CVD prevention cohort over time. DAPT, dual antiplatelet therapy; DA, Disease Analyzer; IMRD, IQVIA Medical Research Data; UK, United Kingdom.

Of the 23,073 patients starting on DAPT in Germany, low-dose aspirin was started in combination with clopidogrel (65%), prasugrel (16%), and ticagrelor (20%). In the UK DAPT cohort, 78% started low-dose aspirin with clopidogrel, 5% with prasugrel, and 17% with ticagrelor. We observed a clear transition from DAPT to low-dose aspirin monotherapy within 2 years of treatment initiation (Figure 4; Supplementary Table 8). This was most notable in the UK, where there was a steep transition from DAPT to low-dose aspirin monotherapy between 300 and 400 days after the index date.

## 4. Discussion

In this large population-based study set in two Western European countries, persistence to low-dose aspirin 10 years after treatment initiation was 35% (Germany) and 47% (UK) among secondary CVD prevention users and was lower at 32% (Germany) and 34% (UK) among primary CVD prevention users. The largest decline in persistence was seen in the first few years following the start of treatment, declining more gradually thereafter. Long-term adherence (between 5 and 10 years) was 60% (Germany) and 75% (UK) among secondary CVD prevention users and was lower at 50% among primary CVD prevention users in both countries.

The estimates from our study are highly valuable and address a data gap in the published literature on long-term (>2–3 years) adherence and persistence. By using two longitudinal healthcare databases, we were able to obtain measures for these outcomes at up to 10-years' follow-up. Among other studies in this area, Filippi et al. [9] reported a 76% 2-year adherence level based on over 45,000 low-dose aspirin users in Italy. An earlier study in Scotland of 17,244 low-dose aspirin users reported 47% adherence over a 6-year study period [14]. Another study from Scotland, based on a secondary CVD prevention population, found that 60% had “good adherence” over an average follow-up of 4.7 years [18]. In a recent database study from France, Ajrouche et al. [13] reported 40% of the 11,793 low-dose aspirin users in the study to be at least 80% adherent at 3 years, as assessed by the PDC. In our study, long-term persistence and adherence to low-dose aspirin were highest in secondary CVD prevention DAPT cohorts. Ten-year persistence in this patient subgroup was 45% in Germany and 75% in the UK, and long-term (5–10 years) adherence was 73% in Germany and 95% in the UK. High levels of low-dose aspirin persistence in patients receiving/eligible for DAPT have been shown previously. In the UK, Saez et al. [15] reported an 85% 1-year persistence level among patients discharged from hospital after ACS using the same database as our study, which is consistent with the 84% 2-year persistence rate seen in our UK secondary CVD prevention DAPT cohort. In Canada, a study by Simpson and colleagues [17] found 1-year persistence to be 71% among 9134 elderly patients using low-dose aspirin after myocardial infarction, based on a definition that required sustained use of the drug for at least 80% of the study period [17]. The higher rates seen in these patients areunderstandable considering the seriousness of an acute coronary event and the more frequent healthcare visits they would require. The clear switch from DAPT to low-dose aspirin monotherapy around a year after the start of treatment, especially in the UK, indicates good compliance with clinical guidelines that advocate a year of DAPT therapy for most patients [2, 3].

Our findings suggest that less than half of patients started on low-dose aspirin for secondary CVD prevention are persistent after 10 years, and that long-term (5–10 years) adherence is also suboptimal, which has important clinical implications considering the chronicity of CVD and the necessity for life-long therapy. Nonadherence and nonpersistence to prophylactic low-dose aspirin therapy are associated with an increased risk of CV events, [10–12, 28] with a 3-fold increased risk of major adverse cardiac events reported in a 2006 meta-analysis [28]. Additionally, in two large population-based studies among users of low-dose aspirin for secondary prevention, Garcia-Rodriguez et al. found that recent discontinuation was associated with a significant 43% increased risk of myocardial infarction [11] and a 40% increased risk of ischaemic stroke [10] compared with current users. Identifying reasons for nonadherence and nonpersistence were beyond the scope of this study, but are likely multifactorial, including patient-related factors such as perception of the seriousness of their illness or education level, medication-related factors such as fear or experience of side effects (including serious conditions such as major bleeding) or high pill burden, and healthcare-related factors such as level of trust in the patient–physician relationship [12, 29, 30]. Our observation of waning adherence to low-dose aspirin over time, especially in primary CVD prevention individuals, is understandable given the challenges of maintaining long-term medication, especially in the absence of previous clinical disease (primary prophylaxis). Aside from the generally higher levels of low-dose aspirin adherence and persistence seen in the UK, we also saw that use of low-dose aspirin for primary CVD prevention was higher in the UK (38% of users) than in Germany (30% of users), and that these users were on average younger in the UK (65 years vs. 70 years).

Key strengths of the study are the addition of novel information to the knowledge base on this topic and the use of standardised methodology to enable a unified analysis across databases from two European countries. Also, as the two data sources are representative of the general population of the respective countries, our findings have good generalisability. We limited our definition of low-dose aspirin to 75–100 mg and excluded patients with a dose of >100–325 mg because this is not a commonly prescribed dose for CVD prophylaxis, while 75–100 mg represents the majority of low-dose aspirin prescriptions in Germany and the UK. Other limitations include the potential for underestimating adherence/persistence due to unrecorded use of over-the-counter low-dose aspirin (especially in the context of primary CVD prevention) and the underestimation of persistence due to potential stockpiling not being taken into account. Also, the data on low-dose aspirin from both data sources reflect prescriptions issued, so we cannot be certain that these prescriptions were always filled and medication subsequently taken, although we believe that returning for repeat prescriptions at regular intervals most likely indicates continuation of therapy. Similarly, in previous research from the US, evaluating the consistent use of secondary CVD prevention therapies, based on follow-up surveys between 1995 and 2002, 71% of patients were using aspirin consistently [31]. Lastly, there may have been a small degree of misclassification among the primary CVD prevention cohort due to the potential for under-recording of CVD conditions in some patients.

In conclusion, long-term adherence and persistence to prophylactic low-dose aspirin is suboptimal. Efforts to encourage persistence with therapy in the first few years of treatment could potentially help longer-term persistence levels and potentially translate into a lower CVD burden in the general population.

## Figures and Tables

**Table 1:** Demographics and median observation time of the study cohorts.

Data source	Cohort	*N*	Mean age (SD), years	% female	Median observation time^∗^ (IQR), years
IQVIA Germany disease analyzer	Primary CVD prevention	43,013	69.5 (12.7)	51	4.3 (2.4–6.8)
	Secondary CVD prevention	101,704	69.0 (12.3)	42	4.1 (2.4–6.5)
	Secondary CVD prevention DAPT^†^	23,073	65.3 (12.3)	32	4.0 (2.4–6.2)

IMRD UK	Primary CVD prevention	72,496	65.0 (15.2)	49	4.8 (2.8–7.3)
	Secondary CVD prevention	118,411	65.5 (13.1)	45	4.4 (2.6–6.8)
	Secondary CVD prevention DAPT^†^	27,648	63.1 (12.7)	31	4.1 (2.4–6.3)

^∗^From the date of the first low-dose aspirin prescription (index date). ^†^Started on DAPT. Abbreviations: CVD, cardiovascular disease; DAPT, dual antiplatelet therapy; IMRD, IQVIA Medical Research Data; IQR, inter-quartile range; SD, standard deviation.

## Data Availability

Data are available from the corresponding author upon reasonable request.

## Abbreviations

CVD: Cardiovascular disease
DAPT: Dual antiplatelet therapy
IMRD: IQVIA medical research data
IQR: Inter-quartile range
SD: Standard deviation.

## Supplementary Materials

Supplementary Materials Supplementary Figure 1. Sensitivity analysis: low-dose aspirin adherence (MPR) within discrete follow-up intervals. Supplementary Figure 2. Sensitivity analysis: low-dose aspirin adherence (MPR) during overlapping time intervals. Supplementary Figure 3. Sensitivity analysis: low-dose aspirin adherence by follow-up time (using PDC) by study cohort. Supplementary Figure 4. Low-dose aspirin persistence over time, stratified by number of consecutive 30 day gaps in therapy. Supplementary Table 1. Type of CVD among the secondary CVD prevention cohort (recorded diagnosis before the first prescription of low-dose aspirin). Supplementary Table 2. Demographics of the primary CVD prevention with risk factors cohort. Supplementary Table 3a. Low-dose aspirin adherence (over patient's entire observation period) by study cohort. Supplementary Table 3b. Low-dose aspirin adherence (over patient's entire observation period) in the primary CVD prevention with risk factors cohort. Supplementary Table 4a. Low-dose aspirin adherence (sensitivity analysis–during distinct periods of follow-up) by study cohort. Supplementary Table 4b. Low-dose aspirin adherence (sensitivity analysis–during distinct periods of follow-up) in the primary CVD prevention with risk factors cohort. Supplementary Table 5. Low-dose aspirin adherence (sensitivity analysis during overlapping follow-up time periods). Supplementary Table 6. Low-dose aspirin persistence over time stratified by consecutive number of ≥60 day gaps. Supplementary Table 7. Low-dose aspirin persistence over time stratified by number of consecutive ≥30 day gaps. Supplementary Table 8. Antiplatelet treatment over time among the secondary prevention DAPT cohort.
