# Supplementary material for: Long-Term Adherence and Persistence to Low-Dose Aspirin for the Prevention of Cardiovascular Disease: A Population-Based Cohort Study
Source: Int J Clin Pract. 2022 Dec 2;2022:7786174. doi: 10.1155/2022/7786174 (PMC9734008; doi:10.1155/2022/7786174)
Supplement: Supplementary Materials — Supplementary Figure 1. Sensitivity analysis: low-dose aspirin adherence (MPR) within discrete follow-up intervals. Supplementary Figure 2. Sensitivity analysis: low-dose aspirin adherence (MPR) during overlapping time intervals. Supplementary Figure 3. Sensitivity analysis: low-dose aspirin adherence by follow-up time (using PDC) by study cohort. Supplementary Figure 4. Low-dose aspirin persistence over time, stratified by number of consecutive 30 day gaps in therapy. Supplementary Table 1. Type of CVD among the secondary CVD prevention cohort (recorded diagnosis before the first prescription of low-dose aspirin). Supplementary Table 2. Demographics of the primary CVD prevention with risk factors cohort. Supplementary Table 3a. Low-dose aspirin adherence (over patient's entire observation period) by study cohort. Supplementary Table 3b. Low-dose aspirin adherence (over patient's entire observation period) in the primary CVD prevention with risk factors cohort. Supplementary Table 4a. Low-dose aspirin adherence (sensitivity analysis–during distinct periods of follow-up) by study cohort. Supplementary Table 4b. Low-dose aspirin adherence (sensitivity analysis–during distinct periods of follow-up) in the primary CVD prevention with risk factors cohort. Supplementary Table 5. Low-dose aspirin adherence (sensitivity analysis during overlapping follow-up time periods). Supplementary Table 6. Low-dose aspirin persistence over time stratified by consecutive number of ≥60 day gaps. Supplementary Table 7. Low-dose aspirin persistence over time stratified by number of consecutive ≥30 day gaps. Supplementary Table 8. Antiplatelet treatment over time among the secondary prevention DAPT cohort. [file 7786174.f1.pdf]

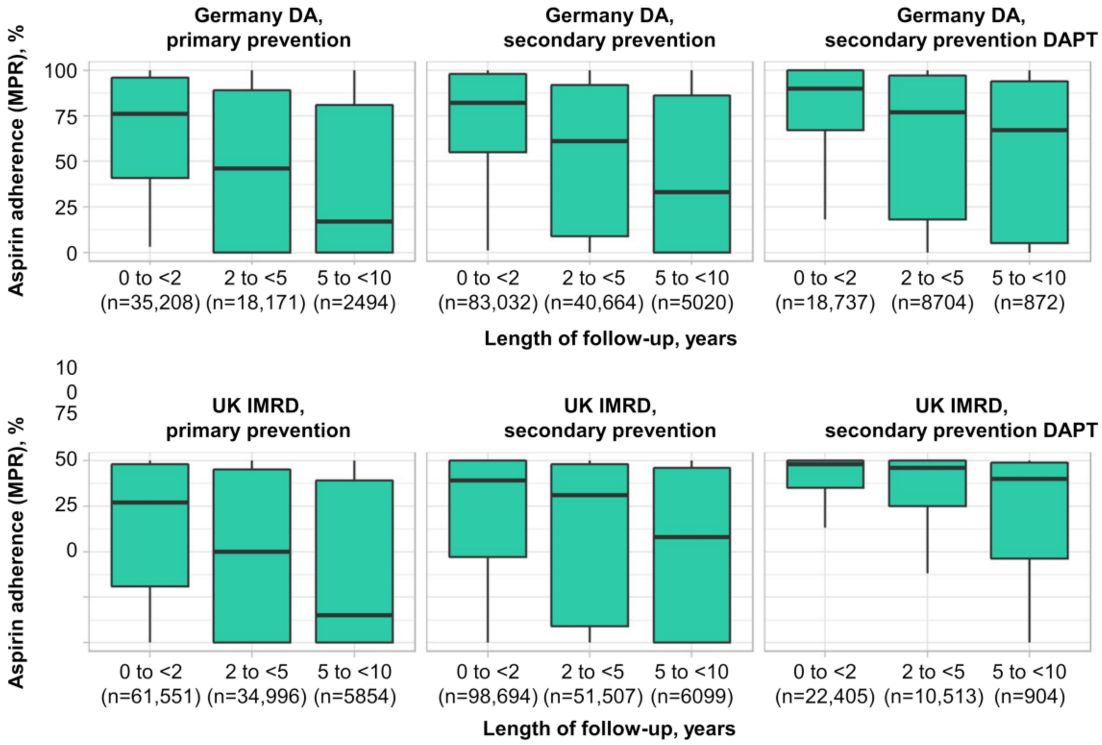

Supplementary Figure 1. Sensitivity analysis: low-dose aspirin adherence (MPR) within discrete follow-up intervals.

DAPT, dual antiplatelet therapy; DA, Disease Analyzer; IMRD, IQVIA Medical Research Data; MPR, medication possession ratio; UK, United Kingdom

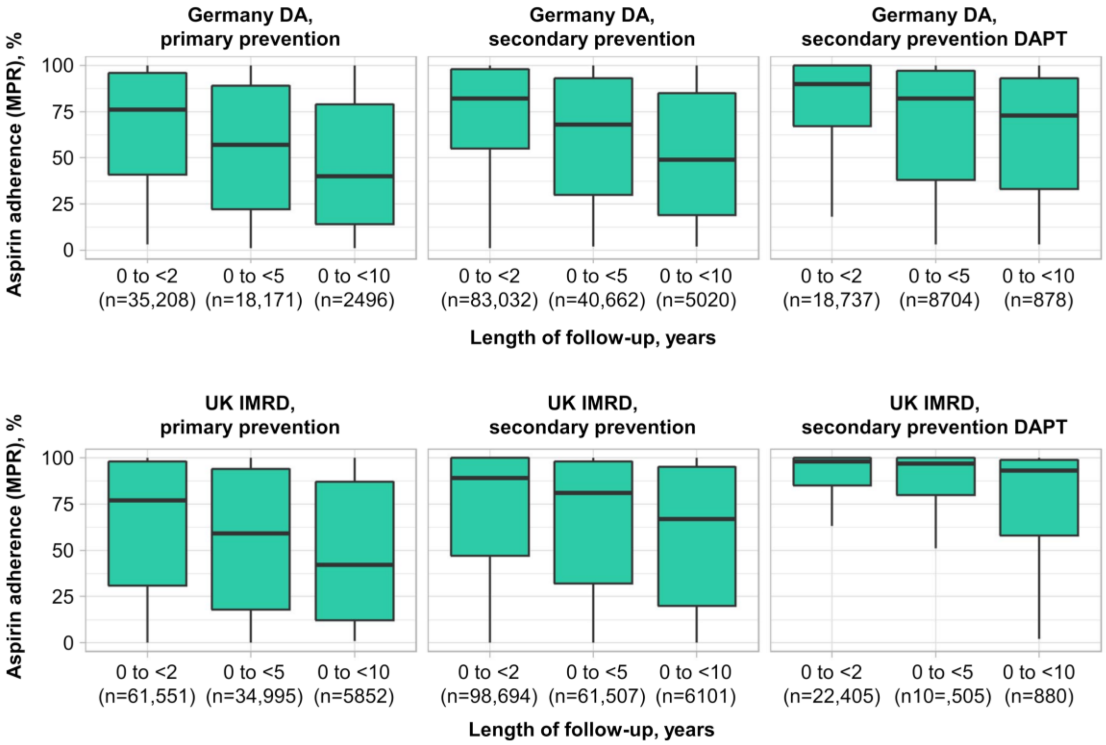

Supplementary Figure 2. Sensitivity analysis: low-dose aspirin adherence (MPR) during overlapping time intervals.
DAPT, dual antiplatelet therapy; DA, Disease Analyzer; IMRD, IQVIA Medical Research Data; MPR, medication possession ratio; UK, United Kingdom

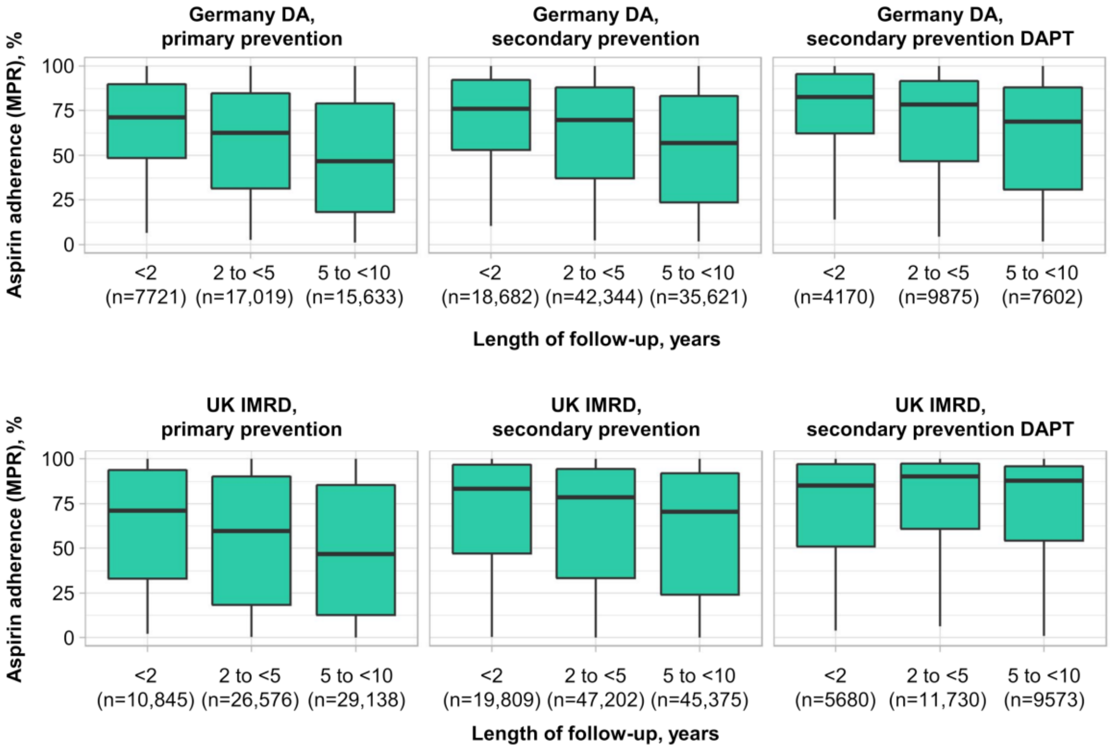

Supplementary Figure 3. Sensitivity analysis: low-dose aspirin adherence by follow-up time (using PDC) by study cohort.

DA, Disease Analyzer; DAPT, dual antiplatelet therapy; IMRD, IQVIA Medical Research Data; UK, United Kingdom

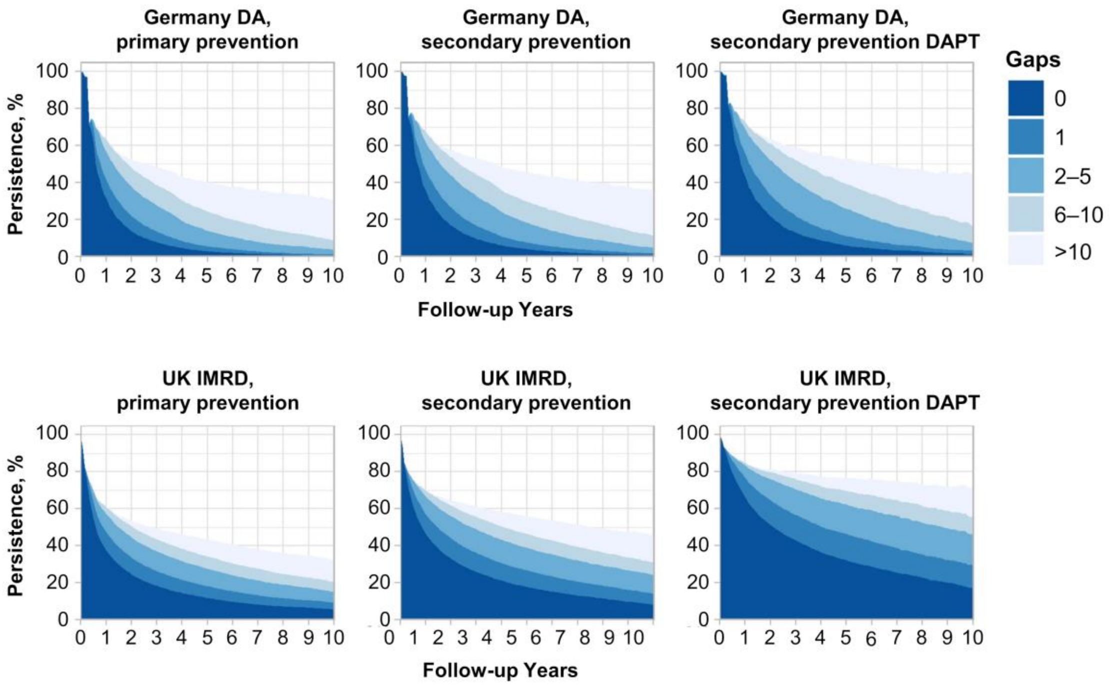

Supplementary Figure 4. Low-dose aspirin persistence over time, stratified by number of consecutive 30 day gaps in therapy.
DAPT, dual antiplatelet therapy; DA, Disease Analyzer; IMRD, IQVIA Medical Research Datal; UK, United Kingdom

Supplementary Table 1. Type of CVD among the secondary CVD prevention cohort (recorded diagnosis before the first prescription of low-dose aspirin).

**Condition (%)**	**IQVIA German Disease Analyzer N=101,704**	**IMRD UK N=118,411**
Acute ischaemic heart disease	14.3	23.8
Angina pectoris	13.5	9.8
Arteriosclerotic vascular disease	34.1	3.4
Cerebral infarction	8.7	1.7
Coronary arteriosclerosis	21.2	3.0
Ischaemic heart disease (other types)	58.5	37.7
Myocardial infarction	20.2	22.6
Coronary thrombosis	10.5	10.9
Transient cerebral ischaemia	10.4	8.5

[*]Recorded any time before the start of follow-up.
Data shown are percentages.
Note: Categories were not mutually exclusive.

CVD cardiovascular disease; IMRD, IQVIA Medical Research Data; UK, United Kingdom

Supplementary Table 2. Demographics of the primary CVD prevention with risk factors cohort.

Data source	N	Mean age (SD), years	% female	Median observation time from first low-dose aspirin prescription (IQR), years
IQVIA German Disease Analyser	41,875	70.3 (11.8)	51	4.3 (2.4–6.8)
IMRD UK	65,090	68.4 (11.9)	46	5.0 (2.8–7.4)

CVD, cardiovascular disease; IMRD, IQVIA Medical Research Data; IQR; inter-quartile range

Supplementary Table 3a. Low-dose aspirin adherence (over patient's entire observation period) by study cohort.

		Median adherence* (IQR), %		
**Source**	**Cohort**	**Patients with <2 years' follow-up**	**Patients with 2 to <5 years' follow-up**	**Patients with 5 to ≤10 years' follow-up**
IQVIA German Disease Analyser IQVIA German Disease Analyser	Primary CVD prevention	75 (50 – 95) (N=7817)	66 (33 – 90) (N=17,035)	50 (19 – 85) (N=15,651)
	Secondary CVD prevention	79 (54 – 97) (N=18,708)	74 (39 – 93) (N=42,360)	60 (25 – 89) (N=35,625)
	Secondary CVD prevention DAPT	86 (65 – 100) (N=4342)	83 (49 – 96) (N=10,035)	73 (32 – 94) (N=7812)
IMRD UK	Primary CVD prevention	76 (34 – 98) (N=10,971)	63 (19 – 95) (N=26,564)	50 (13 – 91) (N=29,132)
	Secondary CVD prevention	88 (50 – 100) (N=19,766)	83 (35 – 98) (N=47,180)	75 (25 – 97) (N=42,372)
	Secondary CVD prevention DAPT	97 (80 – 100) (N=5260)	96 (75 – 100) (N=11,894)	95 (72 – 100) (N=8623)

*Assessed using the MPR.
CVD, cardiovascular disease; DA, Disease Analyzer; DAPT, dual antiplatelet therapy; IMRD, IQVIA Medical Research Data; IQR; inter-quartile range; MPR, medication possession ratio; SD, standard deviation

Supplementary Table 3b. Low-dose aspirin adherence (over patient's entire observation period) in the primary CVD prevention with risk factors cohort.

		Median adherence* (IQR), %		
		**Patients with <2 years' follow-up**	**Patients with 2 to <5 years' follow-up**	**Patients with 5 to ≤10 years' follow-up**
IQVIA German Disease Analyser	N	7602	16,577	15,239
	Adherence	76 (50 – 95)	67 (34 – 90)	50 (20 – 86)
IMRD UK	N	9496	23,371	26,709
	Adherence	83 (44 – 99)	73 (26 – 97)	54 (16 – 92)

*Assessed using the MPR.
DAPT, dual antiplatelet therapy; IMRD, IQVIA Medical Research Datal IQR; inter-quartile range; MPR, medication possession ratio; SD, standard deviation

Supplementary Table 4a. Low-dose aspirin adherence (sensitivity analysis – during distinct periods of follow-up) by study cohort.

		Median adherence* (IQR), %		
**Source**	**Cohort**	**Follow-up years 0 to <2**	**Follow-up years <2 to <5**	**Follow-up years 5 to <10**
IQVIA German Disease Analyser IMRD UK	Primary CVD prevention	76 (41 – 96) (N=35,208)	46 (0 – 89) (N=18,171)	17 (0 – 81) (N=2494)
	Secondary CVD prevention	82 (55 – 98) (N=83,032)	61 (9 – 92) (N=40,664)	33 (0 – 86) (N=5020)
	Secondary CVD prevention DAPT	90 (67 – 100) (N=18,737)	77 (18 – 97) (N=8704)	67 (5 – 94) (N=872)
UK IMRD	Primary CVD prevention	77 (31 – 98) (N=61,551)	50 (0 – 95) (N=34,996)	15 (0 – 89) (N=5854)
	Secondary CVD prevention	89 (47 – 100) (N=98,694)	81 (9 – 98) (N=51,507)	58 (0 - 96) (N=6099)
	Secondary CVD prevention DAPT	98 (85 – 100) (N=22,405)	96 (75 – 100) (N=10,513)	90 (46 – 99) (N=904)

*Assessed using the MPR.
CVD, cardiovascular disease; DA, Disease Analyzer; DAPT, dual antiplatelet therapy; IMRD, IQVIA Medical Research Datal IQR; inter-quartile range; MPR, medication possession ratio; SD, standard deviation

Supplementary Table 4b. Low-dose aspirin adherence (sensitivity analysis – during distinct periods of follow-up) in the primary CVD prevention with risk factors cohort.

	**Median adherence[*] (IQR), %**		
**Source**	**Follow-up years 0 to <2**	**Follow-up years 0 to <2 to <5**	**Follow-up years 5 to <10**
IQVIA German Disease Analyser<br>IMRD UK	77 (41 – 96)<br>(N=34,287)	47 (0 – 89)<br>(N=17,704)	19 (0 – 82)<br>(N=2439)
UK IMRD	82 (38 – 99)<br>(N=55,618)	56 (0 – 96)<br>(N=32,250)	20 (0 – 90)<br>(N=5529)

CVD, cardiovascular disease; IMRD, IQVIA Medical Research Data; IQR, inter-quartile range; UK, United Kingdom

Supplementary Table 5. Low-dose aspirin adherence (sensitivity analysis during overlapping follow-up time periods).

		**Median adherence* (IQR), %**		
**Source**	**Cohort**	**Follow-up years 0 to <2**	**Follow-up years 0 – <5**	**Follow-up years 5 – <10**
IQVIA German Disease Analyser IMRD UK	Primary CVD prevention	76 (41 – 96) (N=35,208)	57 (22 – 89) (N=18,171)	40 (14 – 79) (N=2494)
	Secondary CVD prevention	82 (55 – 98) (N=83,032)	68 (30 – 93) (N=40,664)	49 (19 – 85) (N=5020)
	Secondary CVD prevention DAPT	90 (67 – 100) (N=18,737)	82 (38 – 97) (N=8704)	73 (33 – 93) (N=878)
UK IMRD	Primary CVD prevention	77 (31 – 98) (N=61,551)	59 (18 – 94) (N=34,995)	42 (12 – 87) (N=5852)
	Secondary CVD prevention	89 (47 – 100) (N=98,694)	81 (32 – 98) (N=51,507)	67 (20 – 95) (N=6101)
	Secondary CVD prevention DAPT	98 (85 – 100) (N=22,405)	97 (80 – 100) (N=10,505)	93 (58 – 99) (N=880)

*Assessed using the MPR.
CVD, cardiovascular disease; DA, Disease Analyzer; DAPT, dual antiplatelet therapy; IMRD, IQVIA Medical Research Data; IQR; inter-quartile range; MPR, medication possession ratio; SD, standard deviation; UK, United Kingdom

Supplementary Table 6. Low-dose aspirin persistence over time stratified by consecutive number of ≥60 day gaps.

				Number of gaps in treatment				
**Source**	**Cohort**	**Year**	**Patients (N)**	**None**	**≤1**	**≤5 gaps**	**≤10 gaps**	**Any**
IQVIA German Disease Analyser	Primary CVD prevention	2	35,398	24.9	36.5	49.0	52.8	52.8
		5	18,449	9.0	16.0	27.9	35.0	41.6
		10	2720	2.5	6.0	13.7	19.1	32.1
	Primary CVD prevention with risk factors	1	41,875	43.90	55.90	65.20	65.30	NA
		4	22,739	12.50	21.00	33.60	40.60	44.90
		8	7,052	4.20	8.80	17.80	24.30	35.20
	Secondary CVD prevention	2	83,501	30.4	43.0	55.0	58.3	58.3
		5	41,390	12.0	20.9	33.7	40.9	47.0
		10	5489	3.4	6.9	15.8	23.0	35.2
	Secondary CVD prevention DAPT	2	18,838	38.4	52.3	63.0	65.1	65.1
		5	8887	17.0	28.7	42.8	49.8	54.1
		10	980	5.3	10.0	24.4	33.5	44.5
IMRD UK	Primary CVD prevention	2	61,791	39.7	47.2	53.9	56.2	56.3
		5	35,573	24.4	30.9	38.2	42.1	45.4
		10	6468	14.0	18.5	24.1	28.0	33.8
	Primary CVD prevention with risk factors	1	65,090	56.80	64.10	69.20	69.50	NA
		4	40,012	29.60	36.90	44.50	48.60	50.90
		8	13,799	17.70	23.00	29.30	33.50	38.80
	Secondary CVD prevention	2	99,154	50.7	58.6	65.1	67.4	67.5
		5	52,384	34.9	42.8	50.7	54.8	58.0
		10	6815	22.3	28.8	35.8	40.3	46.8
	Secondary CVD prevention DAPT	2	22,509	71.6	78.8	82.9	84.0	84.0
		5	10,745	57.9	67.5	74.8	78.0	79.8
		10	1016	46.4	57.2	65.6	71.1	75.3

Data shown are percentages.
CVD, cardiovascular disease; IMRD, IQVIA Medical Research Data; NA, not applicable; UK, United Kingdom

Supplementary Table 7. Low-dose aspirin persistence over time stratified by number of consecutive ≥30 day gaps.

				Number of gaps in treatment				
**Source**	**Cohort**	**Year**	**Patients (N)**	**None**	**≤1**	**≤5 gaps**	**≤10 gaps**	**Any**
IQVIA German Disease Analyser	Primary CVD prevention	2	35,398	13.9	22.2	38.1	48.3	52.4
		5	18,449	2.7	5.5	14.2	24.8	40.2
		10	2,720	0.4	1.1	3.8	9.1	30.7
	Secondary CVD prevention	2	83,501	17.0	26.9	44.2	53.9	57.6
		5	41,390	4.0	7.6	18.5	30.1	45.7
		10	5,489	0.8	1.6	4.8	11.9	36.0
	Secondary CVD prevention DAPT	2	18,838	22.0	33.6	52.2	60.8	63.2
		5	8,887	6.0	11.2	26.2	39.6	53.0
		10	980	1.1	3.0	7.7	17.8	45.4
IMRD UK	Primary CVD prevention	2	61,791	24.9	34.0	44.8	50.7	53.6
		5	35,573	11.9	18.3	27.9	34.5	43.6
		10	6,468	5.6	9.3	15.3	20.6	32.5
	Secondary CVD prevention	2	99,154	33.2	44.3	56.3	62.0	64.8
		5	52,384	17.9	26.8	39.1	46.6	56.0
		10	6,815	8.2	14.4	24.5	31.3	46.0
	Secondary CVD prevention DAPT	2	22,509	51.5	64.9	76.2	79.9	81.2
		5	10,745	32.4	46.6	62.5	69.9	76.9
		10	1,016	17.2	29.5	46.4	55.6	71.0

Data shown are percentages.
DAPT, dual antiplatelet therapy; IMRD, IQVIA Medical Research Data; UK, United Kingdom

Supplementary Table 8. Antiplatelet treatment over time among the secondary prevention DAPT cohort.

Source	Years from start of DAPT	DAPT cohort (N)	DAPT (%)	Low-dose aspirin (%)	Other antiplatelet (%)
IQVIA German Disease Analyser	0	23,073	100.0	0	0
	1	23,073*	35.0	35.9	7.4
	2	18,838	9.0	56.4	3.6
IMRD UK	0	27,648	100	0	0
	1	27,648*	48.0	36.2	4.8
	2	22,509	10.0	72.8	4.1

*Equates to the number at the start of follow-up because all individuals were required to have at least 1 year available follow-up.
DAPT, dual antiplatelet therapy; IMRD, IQVIA Medical Research Data; UK, United Kingdom
